# Hydrophobic Modification of Cashew Gum with Alkenyl Succinic Anhydride

**DOI:** 10.3390/polym12030514

**Published:** 2020-02-27

**Authors:** Atanu Biswas, H. N. Cheng, Sanghoon Kim, Carlucio R. Alves, Roselayne F. Furtado

**Affiliations:** 1 National Center for Agricultural Utilization Research, USDA Agricultural Research Services, 1815 N. University Street, Peoria, IL 61604, USA; sanghoon.kim@usda.gov; 2 Southern Regional Research Center, USDA Agricultural Research Service, 1100 Robert E. Lee Blvd., New Orleans, LA 70124, USA; 3 State University of Ceará, Chemistry Department, Silas Munguba Av. 1.700, Fortaleza, CE 60740-020, Brazil; alvescr@yahoo.com; 4 Embrapa Agroindústria Tropical, Rua Dra. Sara Mesquita 2270, Fortaleza, CE CEP 60511-110, Brazil; roselayne.furtado@embrapa.br

**Keywords:** ASA, cashew gum, hydrophobic modification, octenyl succinic anhydride, polysaccharide, tetrapropenyl succinic anhydride

## Abstract

Cashew gum (CG) shows promise of being useful as an agro-based raw material for the production of eco-friendly and biodegradable polymers. In this work, we modified this water-soluble polymer with alkenyl succinic anhydride in order to attach a hydrophobic group to it. The modification used two reagents: octenyl succinic anhydride and tetrapropenyl succinic anhydride. Reactions were conducted at 120 °C using dimethyl sulfoxide as a solvent, with conversions better than 88%. Samples with degrees of substitution (DS) between 0.02 and 0.20 were made. The resulting polymers were characterized using ^1^H NMR, ^13^C NMR, FTIR, TGA, and GPC. The addition of the hydrophobe decreased the affinity of cashew gum for water absorption. Hydrophobically modified polysaccharides are often used as polymeric emulsifiers, thickeners, and compatibilizers; we anticipate that these new hydrophobically modified CGs may be used for the same applications.

## 1. Introduction

Cashew gum (CG) is obtained as an exudate from the bark of the cashew tree (*Anacardium occidentale L.*) [1,2,3]. It is native to Brazil and is cultivated in many countries such as Brazil, India, Nigeria and Kenya. The CG from Brazil is a low-viscosity acidic polysaccharide with the following sugar composition: 72–73% *β*-D-galactose, 11–14% *α*-D-glucose, 4.6–5% *α*-L-arabinose, 3.2–4% *α*-L-rhamnose, and 4.5–6.3% *β*-D-glucuronic acid [1]. It has a highly branched structure, with the main chains comprising 1,3-linked *β*-D-galactopyranosyl units interspersed with *β*-1,6-linked bonds [1,2,3]. It has been used as a thickener, stabilizer, and emulsifier in food formulations; as a binder, excipient, disintegrant, encapsulant, suspension and emulsion agent; even as an active ingredient in pharmaceutical applications [1,2,3]. Several derivatives of CG are known [3,4], but there have been few publications on CG modified with a long alkyl chain (providing strong hydrophobic interactions), except for one report written by us, where alkyl ketene dimer was grafted onto cashew gum with the help of 4-dimethylaminopyridine [5].

A hydrophobically modified polymer typically involves the incorporation of a few hydrophobic groups on a hydrophilic polymer [6]. Such a polymer structure often possesses surfactant-like behavior and desirable rheological characteristics in aqueous polymer solutions and dispersions [6,7,8]. Above a certain polymer concentration, the hydrophobic units associate together, and the solution viscosity increases significantly. The viscosity, however, varies with the shear rate, with lower viscosities observed at higher shear rates, making these materials valuable in paint, personal care, enhanced oil recovery and other applications. Many of these polymers have been commercialized, including hydrophobically modified hydroxyethyl celluloses (HMHECs) [9,10], hydrophobically modified ethoxylated urethanes (HEURs) [11,12], and hydrophobically modified alkali-swellable emulsions (HASEs) [13,14,15]. Being water-soluble biobased polymers, polysaccharides have often been hydrophobically modified, and many such polysaccharides have been reported [5,9,10,16,17,18,19]. 

Alkyl succinic anhydrides (ASAs) constitute a family of relatively inexpensive, commercially available reagents, frequently used as sizing agents for paper [20,21] and as starch modifiers. An ASA is usually synthesized as a product of an ene reaction between an olefin and maleic anhydride, with comparatively few byproducts after reactions [22,23]. Commonly known ASAs include *n*-octenyl succinic anhydride (OSA) and *n*-dodecyl succinic anhydride (DDSA). Derivatives of starch and OSA are known and have recently been reviewed by Altuna et al. [24] and Sweedman [25]. A more general review of modifications with DDSA has been given by Shah [26]. In addition to starch, ASAs have been used to modify other polysaccharides; a summary of selected ASA/polysaccharide derivatives reported in the past 20 years is given in Table 1. The use of ASA for paper sizing applications [20,21], where at least some of the ASA reacts with the cellulose molecules on paper, has not been included in Table 1. 

The purpose of this work was to prepare hydrophobically modified cashew gum using two ASAs: OSA and tetrapropenyl succinic anhydride (TPSA). Previously, one of us [31] had attempted to use dimethylsulfoxide (DMSO) as a solvent for the reaction of starch and OSA. We followed up on this earlier work and used DMSO for the reaction of cashew gum with OSA and TPSA. As far as we know, this is the first report on the ASA derivatives of cashew gum. 

## 2. Materials and Methods

### 2.1. Materials

Gum exudate from the cashew tree was collected from Embrapa Experimental Station at Pacajus (Fortaleza-Ceará). It was ground to 100-mesh particle size, dissolved in water, centrifuged at 10,000 rpm at 4 °C for 20 min, and filtered to remove insoluble materials. Water/ethanol mixture at 1:4 (v/v) ratio was added to the solution for 24 h to precipitate the polysaccharides. The precipitate was filtered, washed exhaustively with acetone, and dried in a hot air circulation oven. The final material is called CG in this work. Both ASA samples were purchased from Milliken Chemical Company (Spartanburg, SC, USA). Other chemicals were acquired from Sigma-Aldrich (St. Louis, MO, USA).

### 2.2. Synthesis of ASA–CG

The CG was dried overnight in a vacuum oven at 70–80 °C before use. Typically, 1 g of CG and 0.02–0.2 g of ASA were dissolved in 4 mL DMSO and heated at 120 °C for 3–6 h in a Reacti-Therm^TM^ setup (Fisher Scientific, Pittsburgh, PA, USA) with constant stirring. The reaction mixture was then cooled down, and isopropanol was added dropwise with stirring to prevent any large clumps from forming. The precipitate was washed with isopropanol and then dried under vacuum at 50–75 °C overnight. 

### 2.3. Characterization Methods

For NMR analysis, each CG–ASA derivative was dissolved in d_6_-DMSO in an NMR tube at a concentration of 10% or higher. The ^1^H and ^13^C NMR spectra were acquired on a Bruker DRX 400 spectrometer (Karlsruhe, Germany) at ambient temperature using standard operating conditions. The chemical shifts were referenced to tetramethylsilane at 0 ppm.

Fourier transform infrared (FTIR) spectra were acquired on a Nicolet iS10 spectrometer (Thermo Scientific Inc., Waltham, MA, USA) equipped with a Smart Orbit single bounce ATR accessory with a diamond crystal. For each spectrum, 32 scans were collected at room temperature at a spectral resolution of 4 cm^−1^ between 600 and 4000 cm^−1^ using a DTGS detector and KBr beam splitter. Data were processed with the Omnics software (version 9.2.98).

Thermogravimetric analysis (TGA) was conducted on a Model Q500 analyzer (TA Instruments, New Castle, DE, USA). Each sample (~5 mg) was weighed into a tared, open platinum TGA pan. The samples were analyzed in a nitrogen atmosphere by heating at 10 °C/min up to 600 °C. 

Size-exclusion chromatography (SEC) was performed using a Shimadzu Prominence LC system (Kyoto, Japan) equipped with refractive index (RI) and diode-array (UV) detectors. DMSO was employed as the mobile phase and solvent for the samples. A 0.5% solution of each sample was prepared in DMSO. Each sample was filtered using a 0.45 μm syringe filter; 50 μL of sample was then injected into the SEC system at a flow rate of 0.5 mL·min^−1^, using a Phenogel 5 μm Linear SEC column (Phenomenex, Torrance, CA, USA) at 60 °C. Molecular weight calibration was carried out with three dextran standards (4, 24, and 100 kDa) and glucose (Mw 180). Thus, the molecular weight range covered was from 180 to 100 kDa.

## 3. Results and Discussion

### 3.1. ASA Reactions

**Scheme 1:**
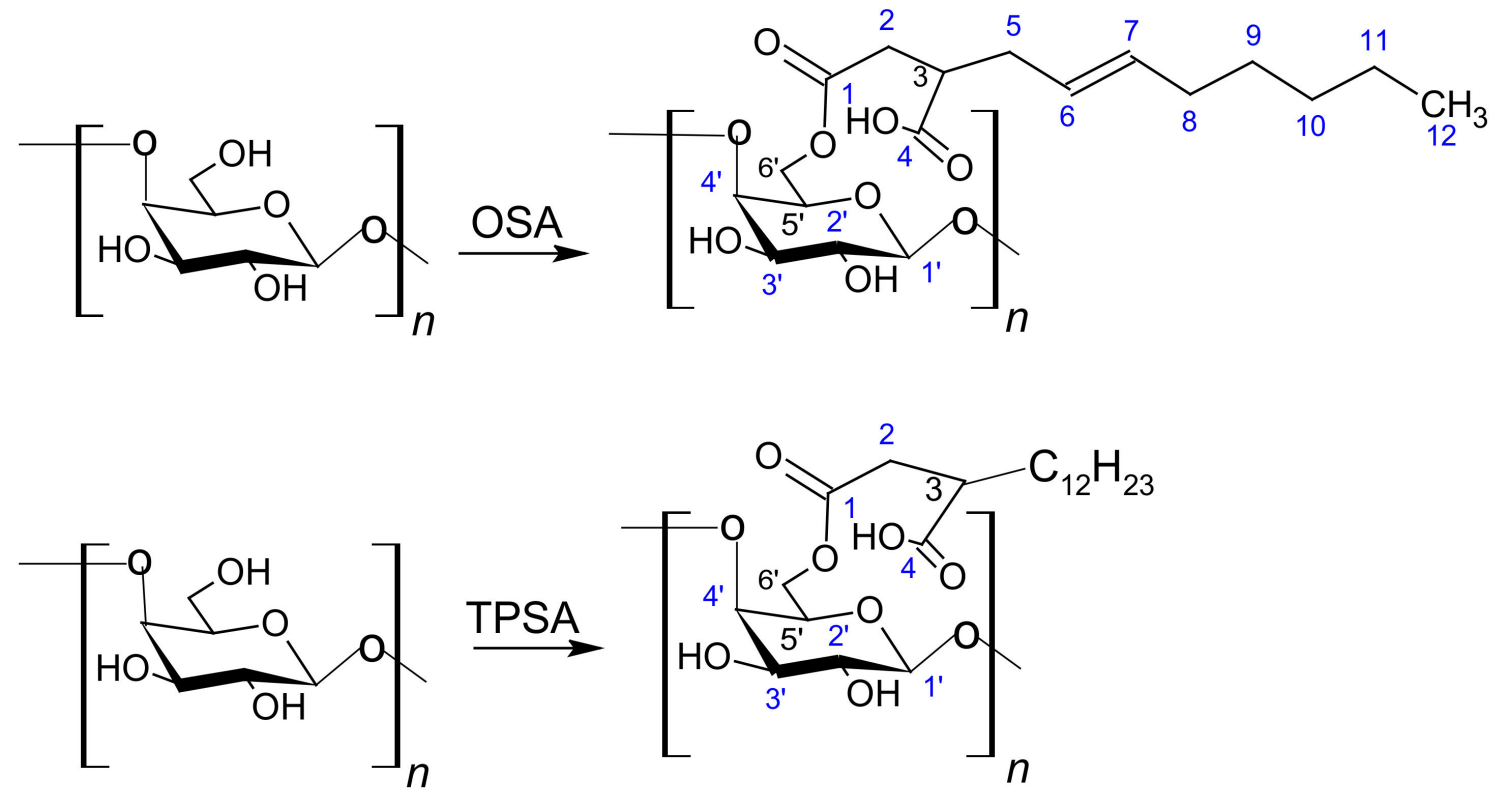
Schematic reaction of cashew gum with OSA (top) and TPSA (bottom). Cashew gum actually contains several sugars; for convenience, only galactose is shown because it is the main component. Substitution of OSA and TPSA can occur at OH positions 2′, 3′, and/or 6′. Only substitution at 6′–OH is shown. On the succinic moiety, the alkyl group can be at position 2 or 3; only position 3 is shown.

**Scheme 2:**
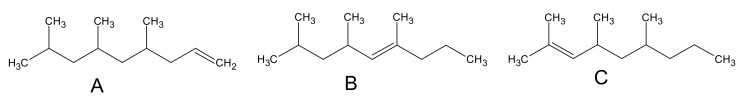
Three examples of propylene tetramer structure.

The reactions of cashew gum with OSA and TPSA are shown schematically in Scheme 1. The structure of the OSA reaction product is straightforward; the OSA substitution can occur at positions 2′, 3′, and/or 6′ on the sugar structure. For TPSA, the structure is much more complex because it is derived from propylene tetramer, which is a mixture of different isomers. Three examples of the propylene tetramer structure are shown in Scheme 2; structure A has a terminal olefin, and structures B and C have internal olefins. Because the ene reaction can take place at either carbon of the double bond [22,23], each structure can give rise to at least two ene products. Thus, a mixture of a large number of TPSA structures is possible; the resulting product of cashew gum and TPSA contains a large number of structures as a result. For convenience, the propylene tetramer structure is abbreviated as C_12_H_23_ in Scheme 1. (In the trade literature [49], sometimes TPSA is also called “dodecenyl succinic anhydride.” We prefer to use TPSA to avoid confusion with the linear *n*-dodecenyl counterpart.)

In most of the prior preparative procedures shown in Table 1, the polysaccharide was dispersed in slightly alkaline water, and the anhydride (in water or ethanol) was added to initiate the reaction. Because of the relatively low solubility of ASA in water, the reaction tended to occur more quickly on the surface of the polysaccharide, resulting in suboptimal reaction efficiency and uneven distribution of reacted groups [25]. In view of our earlier experience with DMSO [31], we chose DMSO for this work and found it to be an easy solvent to work with, and homogeneous products were obtained. The reaction was found to be rather facile at 120 °C, with a 3 h reaction time. The weight yields were all greater than 88% (Table 2). 

### 3.2. Spectroscopic Characterization

**Figure 1:**
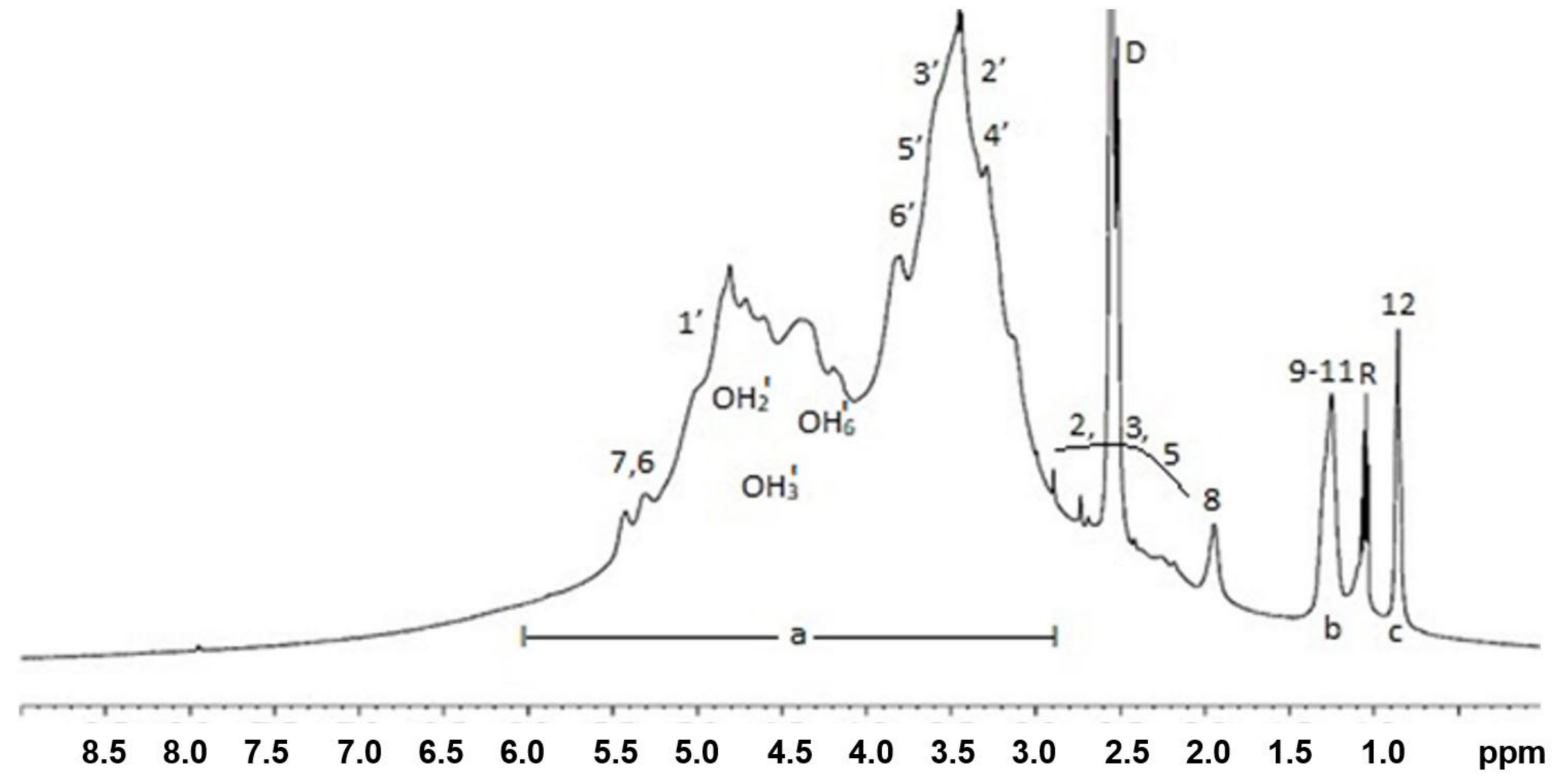
^1^H NMR spectrum of a CG–OSA adduct in d_6_-DMSO. The proton numbers follow the scheme shown in Scheme 1; D = dimethylsulfoxide (DMSO); R = methyl from rhamnose.

The ^1^H NMR spectrum of a CG–OSA product is shown in Figure 1. The CG contained multiple sugar residues, and their peaks can be found under the broad spectral pattern at ca. 2.7–6.0 ppm. The protons attached to the C1s of the sugars occurred at the downfield region, together with the hydroxy groups (4.2–5.5 ppm) [50,51]. The peak at 1.1 ppm is due to the methyl in rhamnose, one of the sugars present in CG. The peaks at ca. 0.8 ppm (peak area c) correspond to the CH_3_ in the OSA residue (position 12 in Scheme 1, top), and the peak at 1.3 ppm (peak area b) comes from CH_2_ at positions 9–11 of the OSA residue [25,39]. Since there were six protons at positions 9–11 and three methyl protons in the OSA residue, the area per mole is b/6 or c/3. The peak area at 2.9–6.0 ppm (a) corresponds roughly to 10 protons on CG sugar residues plus two olefin protons from OSA; thus, the area per mole of CG is (a − 2c/3)/10 or (a − b/3)/10. An estimate of the degrees of substitution (DS) is therefore (b/6)/{(a − b/3)/10} or (c/3)/{(a − 2c/3)/10}. The expected and the observed (estimated) DS values are shown in the last two columns of Table 1. The agreement between these values is satisfactory in view of the errors in determining a small number relative to a large number. 

**Figure 2:**
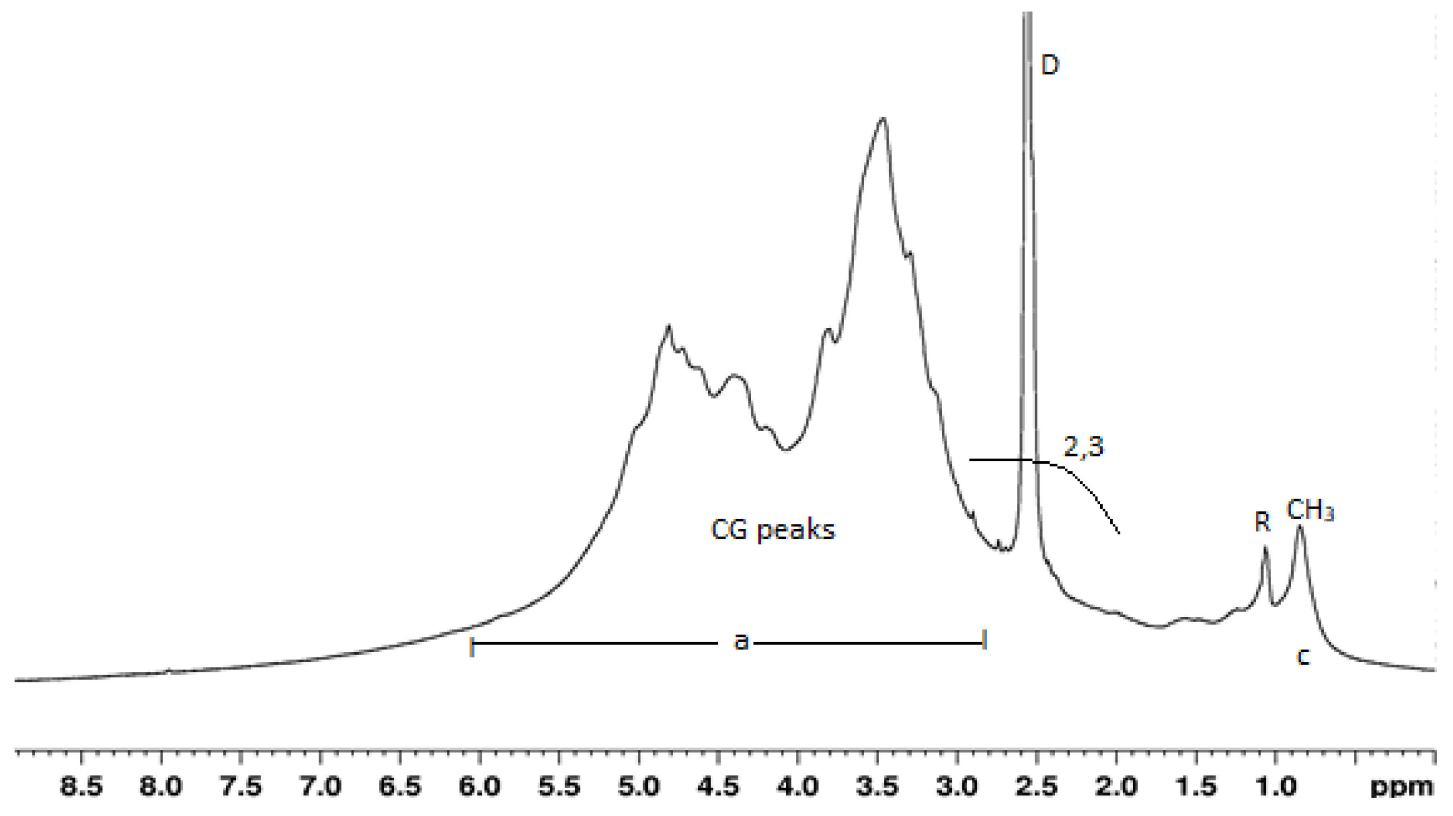
^1^H NMR spectrum of a CG–TPSA adduct in d_6_-DMSO. The proton numbers for succinate follow the scheme shown in Scheme 1; D = DMSO; R = methyl from rhamnose.

The ^1^H NMR spectrum of a CG–TPSA product is shown in Figure 2. As before, the CG peaks can be found at ca. 2.9–6.0 ppm (peak area a). In view of the complexity of the TPSA structure, we chose to use methyl peak at 0.8 ppm for the calculation (peak area c). Since there are 12 protons in the four (non-allylic) methyl groups, the area per mole for the TPSA residue is c/12. The olefin protons in TPSA show up at around 4.9–5.3 ppm, in the same region as the CG peaks. Thus, we need to subtract one olefin proton (c/12) from the area of CG peaks. The area per mole for CG is then (a − c/12)/10. The DS can be estimated via the following expression: (c/12)/{(a − c/12)/10}. The expected and the estimated DS values are shown in the last two columns of Table 1. The agreement between these values is again satisfactory. 

**Figure 3:**
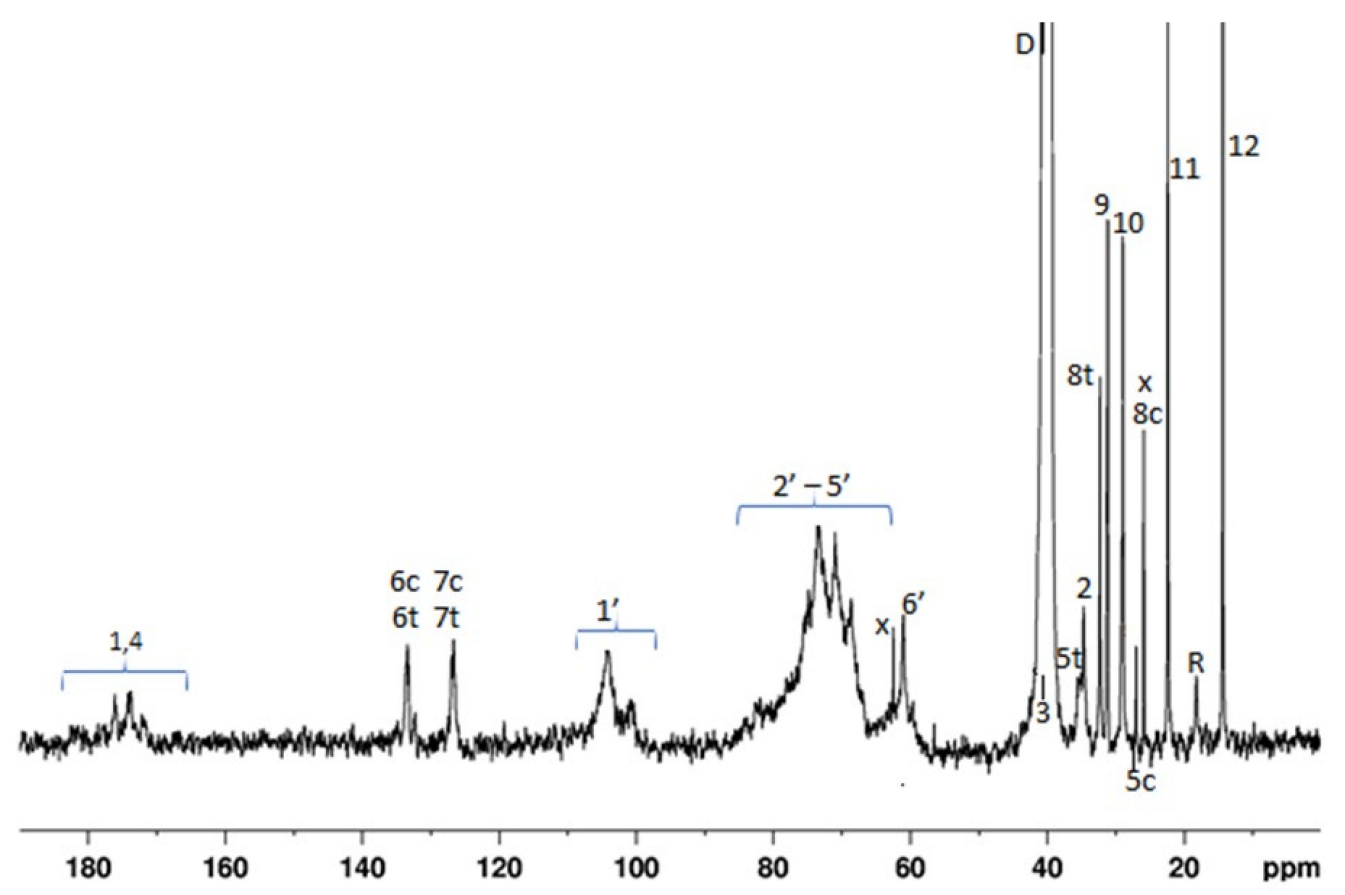
^13^C NMR spectrum of a CG–OSA adduct in d_6_-DMSO. The carbon numbers follow Scheme 1; D = DMSO; R = methyl from rhamnose, x = isopropanol, c = cis, t = trans.

**Figure 4:**
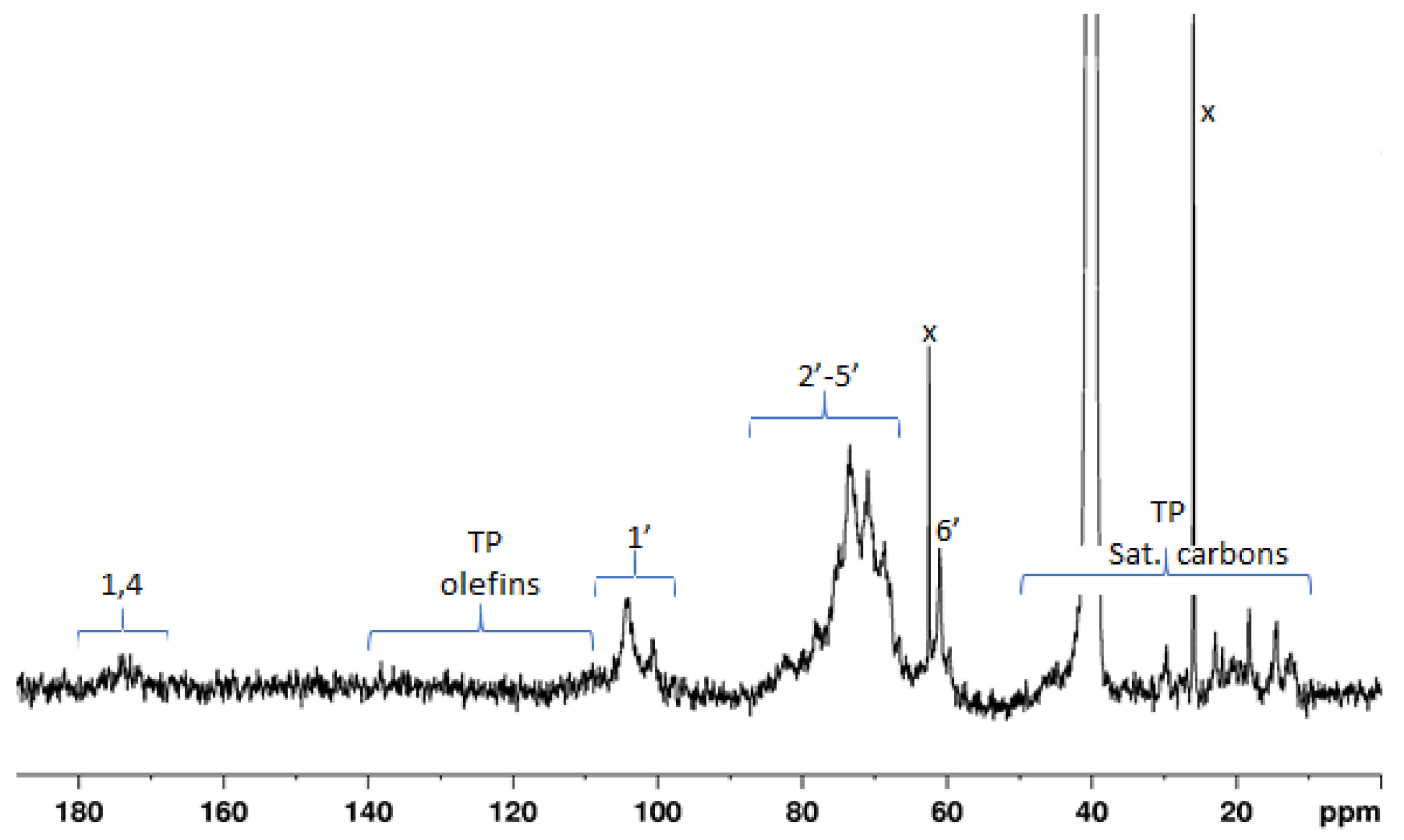
^13^C NMR spectrum of a CG–TPSA adduct in d_6_-DMSO. The carbon numbers follow Scheme 1; D = DMSO; R = methyl from rhamnose, x = isopropanol, TP = tetrapropene moiety.

Further confirmation of the structures of CG derivatives was obtained from ^13^C NMR (Figure 3 and Figure 4). The ^13^C NMR assignments for CG were previously reported [52,53] and were compatible with the general assignments of polysaccharides [50]. The CG sugar residues resonated at 58–110 ppm, with C1 at ca. 100–108 ppm, C6 at ca. 58–66 ppm, and C2–C5 at ca. 65–87 ppm (Figure 3). The ^13^C NMR spectrum of starch-OSA product was previously assigned by Bai et al. [39]. We have adapted their assignments of the OSA moiety to the CG–OSA product, as shown in Figure 3. Note that the ester and carboxyl carbons can be clearly seen at 170–180 ppm; the multiple peaks observed in this region are due to the different positional isomers possible for substitution of OSA at positions 2′, 3′, and/or 6′ at CG, and regioisomers due to alkyl placement on position 2 or 3 of the succinic moiety (Scheme 1). 

In the ^13^C NMR spectrum of the CG–TPSA adduct (Figure 4), the CG carbons can be observed at roughly the same ^13^C shifts as in Figure 3. However, because of the complex nature of the propylene tetramer (TP) structures, multiple peaks are observed in the 14–46 ppm region for the saturated carbons of the TPSA, and specific structural assignments cannot be made. Note that in the 170–180 ppm region, again, ester and carboxyl peaks are found, confirming the ester linkage between CG and TPSA. 

**Figure 5:**
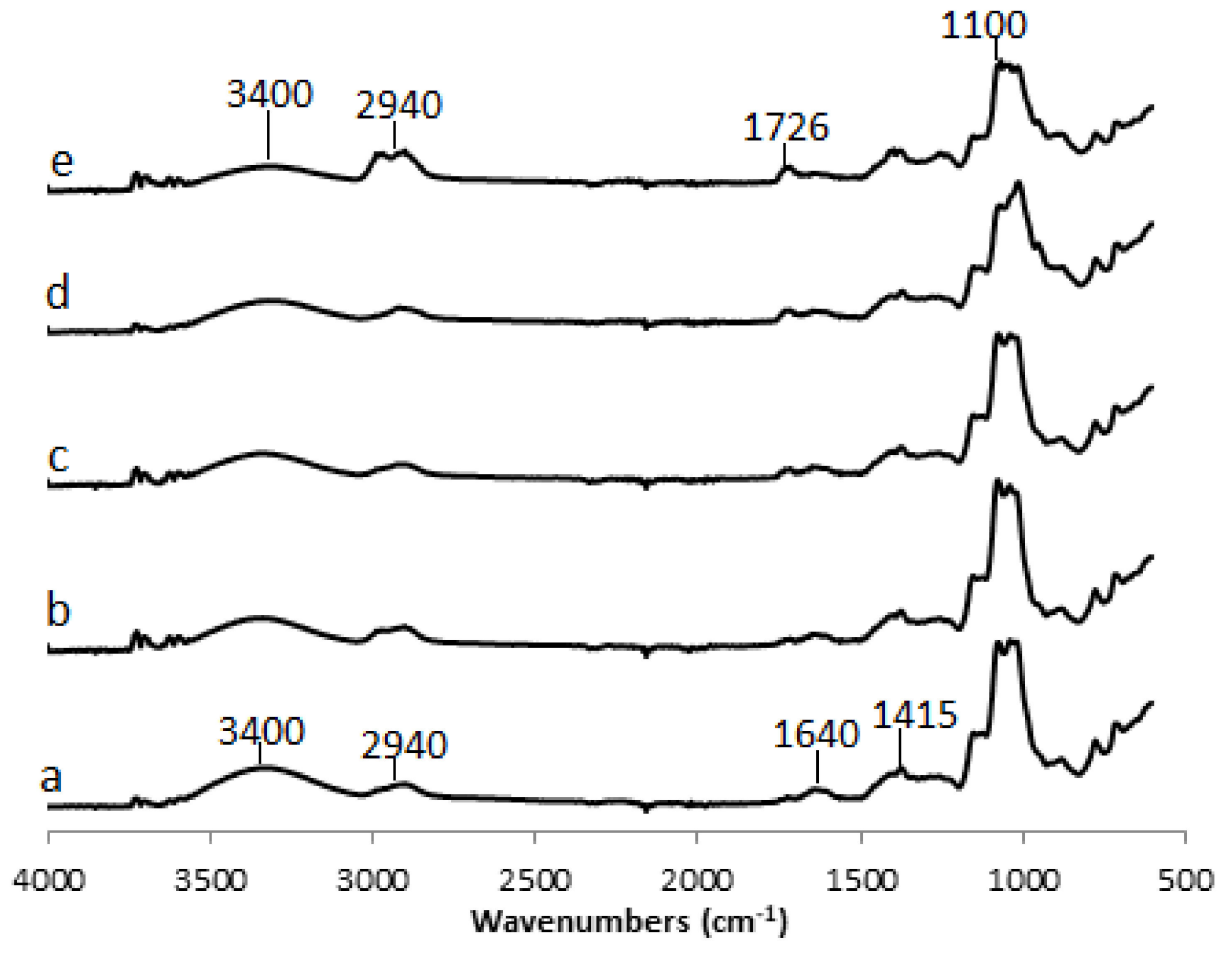
FTIR spectra for CG–OSA adducts from the bottom to the top: **a**) sample O1, **b**) sample O2, **c**) sample O3, **d**) sample O4, and **e**) sample O5.

The FTIR spectra of the CG–OSA adducts (samples O1–O5) are shown in Figure 5. The spectra exhibit the bands that are characteristic of polysaccharides [51,53,54,55], viz., large bands at ca. 1100 cm^−1^ for C–O and C–C vibrations from the pyranose ring, a broad O–H stretching band at ca. 3400 cm^−1^, C–H vibration peaks at about 2940 cm^−1^, O–H bending at 1415 cm^−1^, and 1640 cm^−1^ for water bound to the polysaccharide. With increasing amounts of OSA reaction, a peak at ca. 1726 cm^−1^ becomes increasingly prominent due to ester formation. The carboxylic acid peak appears at 1700–1720 cm^−1^, overlapping the ester peak, as shown earlier in the FTIR spectra of inulin succinate [56] and viscose rayon succinate [57]. With increasing OSA reaction, the C–H vibration at ca. 2940 cm^−1^ also becomes larger, whereas the broad O–H stretching band at 3400 cm^−1^ gets smaller. These observations corroborate the NMR results with respect to the reaction of OSA with cashew gum. 

**Figure 6:**
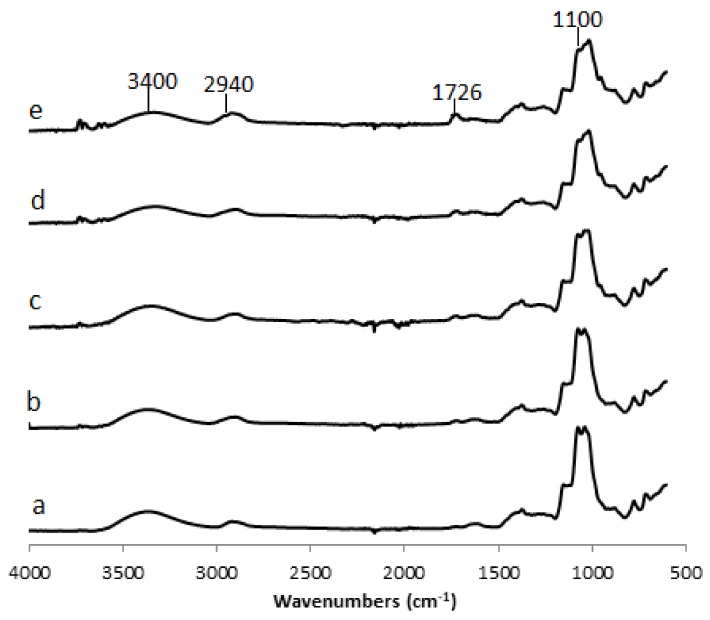
FTIR spectra for CG–TPSA adducts from the bottom to the top: **a**) sample T1, **b**) sample T2, **c**) sample T3, **d**) sample T4, and **e**) sample T5.

The FTIR spectra of the CG–TPSA adducts (samples T1–T5) are shown in Figure 6. The observation here is similar to that for the CG–OSA adducts. As before, the spectral features attributable to cashew gum can be clearly observed. With increasing TPSA reaction, both the ester peak at 1726 cm^−1^ and the C–H stretching at ca. 2940 cm^−1^ became increasingly prominent, thus confirming the structure of the desired reaction product.

### 3.3. Thermogravimetric Analysis

**Figure 7:**
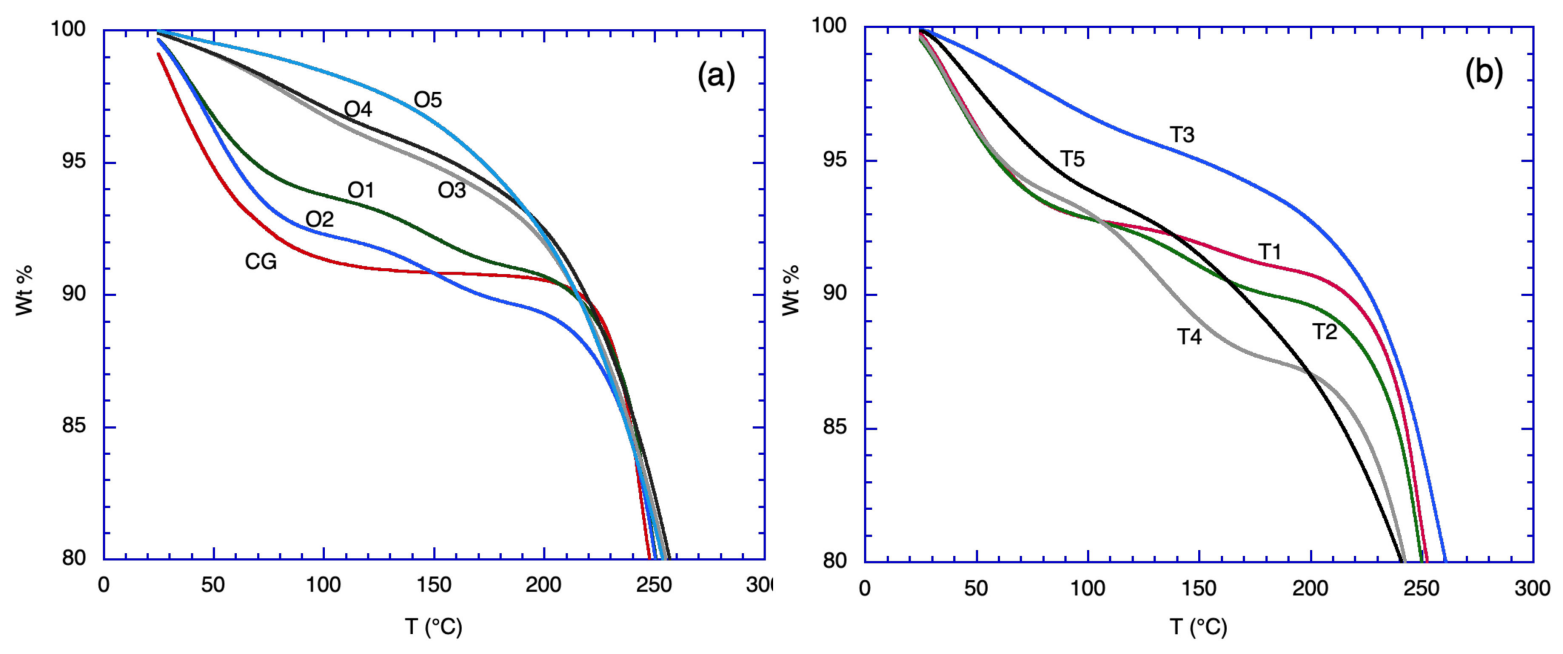
TGA data for CG–OSA adducts (left) and CG–TPSA adducts (right). Sample numbers are the same as given in Table 2.

The TGA data for the CG–OSA adducts are shown in Figure 7 (left plot). The curve for non-modified CG shows a rapid loss of water (about 9% by weight) at 50–100 °C, followed by a significant weight loss at around 250 °C due to incipient polysaccharide degradation. This result is consistent with prior reports on the TGA of CG [53,54,58]. With OSA modification, the weight loss of water decreases, suggesting that the hydrophobic OSA modification reduces the affinity of CG to water, such that less water was found in modified CG. The situation is slightly complicated by the incipient breakage of ester bonds between CG and OSA and the release of alkyl chains, which starts at about 219 °C [45]. Thus, samples O1 and O2 show slightly lower weight loss at about 200 °C than expected. 

The TGA data for the CG–TPSA adducts are given in Figure 7 (right plot). If we compare the weight loss of water at 50–100 °C for samples T1–T5 versus the CG control curve on the left plot, all the hydrophobically modified CGs show reduced weight loss. However, there is no clear trend of increasing TPSA modification versus water loss. In particular, samples T4 and T5 start to lose more weight than CG controls at 150–250 °C, indicating the breakage of ester bonds between CG and TPSA and the release of the alkyl chains. Because of the complex and heterogeneous nature of TPSA, perhaps some components of TPSA may be more amenable to ester degradation and volatilization at 150–250 °C.

### 3.4. Molecular Weight Distribution

**Figure 8:**
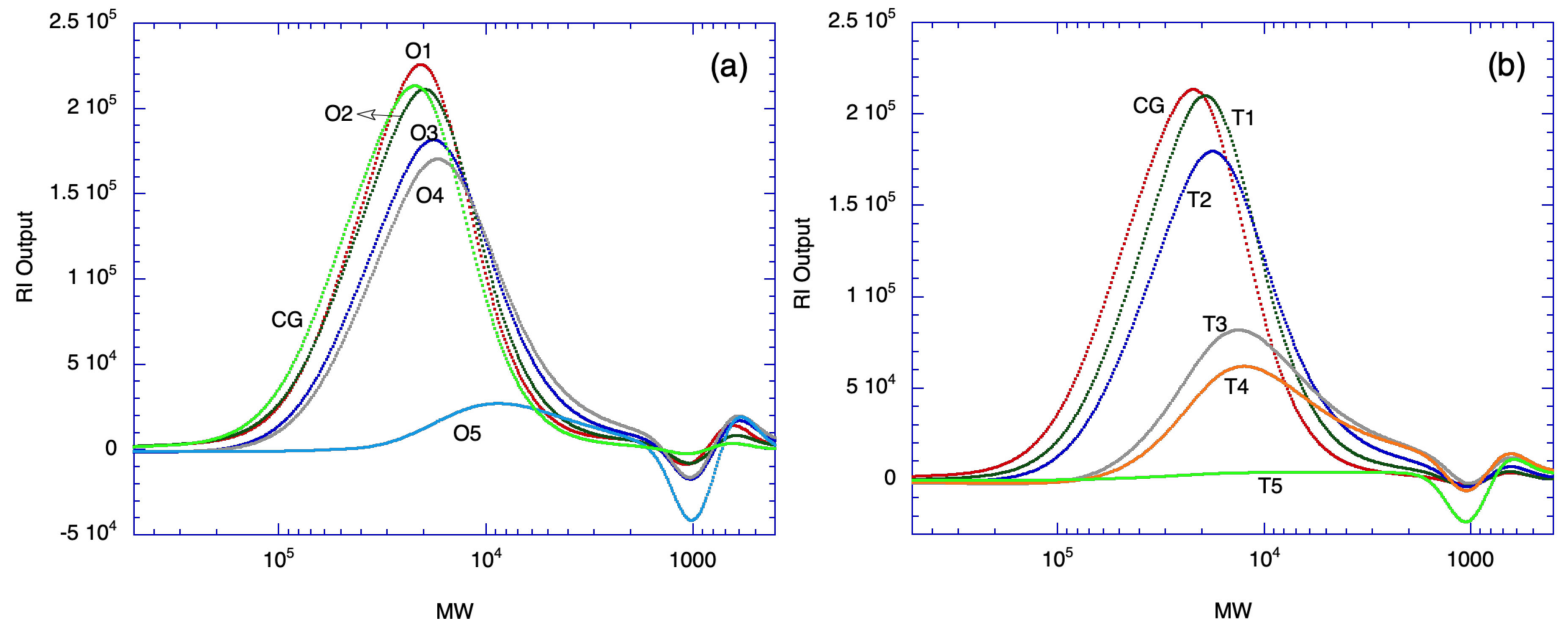
GPC data for CG–OSA adducts (**a**) and CG–TPSA adducts (**b**). Sample numbers are the same as given in Table 3.

It is useful to determine the molecular weight distribution of the unmodified and modified CG via gel permeation chromatography (GPC). The GPC curves for the CG–OSA adducts are shown in Figure 8 (left plot). For unmodified CG, Mn = 18.2 kDa, and Mw = 31.7 kDa (Table 3). Previously, Neto et al. reported a peak molecular weight of 23,000 kDa [59] and Silva et al. reported Mn 19,700 kDa and Mw 23,500 kDa [60] for CG. Thus, our result is comparable to what was reported before. With increasing OSA modification, the GPC curve shifts slightly to lower molecular weights. However, the intensity of the GPC peak also decreases, indicating that part of the CG–OSA polymer is aggregated and/or retained on the column. The effect is particularly noticeable for sample O5, where the peak intensity drops significantly, indicating that most of the polymer is not detectable, and whatever is detected is only the lower-molecular-weight fraction. It is well known that hydrophobically modified polymers can undergo aggregation in GPC analysis [61,62]. Thus, it is not surprising that hydrophobically modified CGs exhibit aggregation that affects the GPC elution behavior of the polymers.

The GPC curves for the CG–TPSA adducts are given in Figure 8 (right plot). The situation with TPSA is similar to with OSA, except that the aggregation/retention phenomenon is more severe. Again, samples T1 and T2 show slightly reduced peak intensities versus unmodified CG with somewhat lower molecular weights (Table 3). Samples T3 and T4 exhibit considerably reduced peak intensities and even lower molecular weights. In the case of sample T5, most of the sample is aggregated and retained on the GPC columns. Thus, the results suggest that CG–TPSA shows more aggregation than CG–OSA. 

In the literature, CG has been used for food, drug, and personal care applications [1,2,3]. Moreover, the hydrophobic modification of polysaccharides is useful for imparting special properties to the polysaccharides, such as emulsification, viscosity improvement and additive compatibility [7,8,16,17]. Thus, it seems reasonable to use hydrophobically modified CG for these same applications, particularly in food, drug, and personal care areas. 

## 4. Conclusions

Agricultural processes typically involve huge amounts of agricultural products, byproducts, and waste materials every year. These materials are available, relatively inexpensive, non-hazardous, biodegradable, and sustainable; thus, they represent good opportunities for the development of value-added derivatives for a variety of applications. This work is part of our efforts to modify cashew gum (one of the agro-based materials) with two hydrophobic reagents in the ASA family. CG–ASA samples with degrees of substitution up to 0.20 were made, and their chemical structures were verified with NMR and FTIR. The thermal properties and molecular weights were studied with TGA and GPC, respectively. These new polymers should be good additions to the family of hydrophobically modified polysaccharides that have been previously reported. From the current study, we know that hydrophobically modified CGs show reduced water content and greater propensity for aggregation in solution relative to CG. Further studies will be needed in the future to further explore their properties and to assess possible applications for these new polymers.

## Figures and Tables

**Table 1:** Selected polysaccharides’ ASA derivatives. ASAs used included *n*-octenyl succinic anhydride (OSA), *n*-dodecyl succinic anhydride (DDSA), and tetrapropenyl succinic anhydride (TPSA).

Substrate	ASA used	Solvent used	Reference
corn starch	DDSA	water suspension, pH 8.5–9.0	[27]
corn starch	OSA	pyridine	[28]
corn and amaranth starch	OSA	pyridine	[29]
corn starch	OSA	glacial acetic acid, 180 °C	[30]
corn starch	OSA	pyridine, DMSO, microwave	[31]
corn starch	DDSA	water suspension, pH 8.5	[32]
corn starch	DDSA	starch film dipped in ethanol-diluted DDSA	[33]
cassava starch	OSA	water, microwave	[34]
corn starch	OSA	water suspension, pH 8.5	[35]
rice amylodextrin	OSA	starch/OSA dissolved in water and dried; heated to 130–150 °C	[36]
corn starch	OSA	water suspension, pH 8.5	[37]
corn starch	OSA	pyridine	[38]
corn starch	OSA	water suspension, pH 7.5–9.5	[39]
inulin	OSA, DDSA	water suspension	[40]
inulin	OSA, DDSA	water dispersion, pH 8.5	[41]
gum arabic	DDSA	water dispersion pH 8.3	[42]
gum arabic	OSA	water dispersion	[43]
corn starch	DDSA	aqueous solution, extruded	[44]
gum karaya	DDSA	water dispersion, pH 8.5	[45]
gum arabic	OSA	water dispersion, pH 8	[46]
basil seed gum	OSA	water dispersion, pH 8	[47]
guar gum	DDSA	water-NaHCO_3_	[48]
cashew gum	OSA, TPSA	DMSO, 120 °C	this work

**Table 2:** Reactions of 1 g cashew gum (CG) with alkenyl succinic anhydride (ASA) in 4 mL DMSO at 120 °C with a 3 h reaction time.

Sample	ASA used	Weight of ASA, g	Product weight, g	Yield %	Expected DS	Obsd DS
O1	OSA	0.02	0.955	94	0.015	0.021
O2	OSA	0.04	0.972	93	0.031	0.035
O3	OSA	0.084	1.054	97	0.065	0.063
O4	OSA	0.104	1.039	94	0.080	0.074
O5	OSA	0.2	1.063	89	0.154	0.155
T1	TPSA	0.02	0.930	91	0.012	0.012
T2	TPSA	0.04	0.941	90	0.024	0.024
T3	TPSA	0.084	1.034	95	0.051	0.048
T4	TPSA	0.104	0.987	89	0.063	0.062
T5	TPSA	0.2	1.050	88	0.122	0.113

**Table 3:** Number-average (Mn) and weight-average (Mw) molecular weights for CG and modified CGs.

Sample	ASA used	Mw (kDa)	Mn (kDa)	Mw/Mn
CG control	none	31.7	18.2	1.74
O1	OSA	28.5	16.3	1.75
O2	OSA	28.1	15.4	1.83
O3	OSA	23.2	12.5	1.86
O4	OSA	21.4	11.3	1.89
O5	OSA	10.0	6.1	1.64
T1	TPSA	26.1	14.4	1.81
T2	TPSA	22.4	11.7	1.91
T3	TPSA	14.4	7.3	1.97
T4	TPSA	13.2	6.8	1.94
T5	TPSA	12.3	5.4	2.28
